# Detection and destruction of antibody-bound viruses by TRIM21

**DOI:** 10.1186/1742-4690-10-S1-O38

**Published:** 2013-09-19

**Authors:** William McEwan

**Affiliations:** 1 MRC Laboratory of Molecular Biology, Cambridge, UK

## 

The tripartite motif family of proteins are emerging as key players in defence against viral infection. The best-characterised member is TRIM5, which possesses the ability to both restrict infection and initiate innate immune detection following infection by retroviruses. We identify TRIM21 as a sensor for cytoplasmic antibody-bound pathogens. Detection of intracellular antibody-bound pathogens leads to rapid proteasome-mediated destruction of virus capsids. Furthermore, TRIM21 is able to synthesise K63-linked ubiquitin chains resulting in signalling via NF-kB, AP-1 and IRF3/5/7 pathways. Detection by TRIM21 is sufficient to induce the production of cytokines including CXCL 10, lL-6 and IFN-Beta. A wide range of intracellular pathogens, including intracellular bacteria, DNA and RNA viruses can be detected by TRIM21. However, enveloped viruses, including retroviruses are able to evade detection by TRIM21 by shedding antibodies during membrane fusion. This study demonstrates that TRIM21 joins TRIM5 in an emerging class of protein that simultaneously provide virus detection and destruction activities.

